# Biology Open: The story so far…

**DOI:** 10.1242/bio.201511643

**Published:** 2015-01-08

**Authors:** Jordan W. Raff, Rachel Hackett

It is hard to believe that, with the arrival of 2015, Biology Open (BiO) begins its fourth year of publication. We'd like to take this moment to look back on what we have learned since the first issue was published by The Company of Biologists in January 2012.

BiO was launched with the aim of reducing the ‘pain to publish’ that many authors experience. By publishing all papers that address a valid scientific question, are technically sound, and where the conclusions are adequately supported by the data, BiO provides a welcoming home for solid, well-executed research papers. We have helped authors to avoid additional rounds of submission and review for papers described as “interesting, but no new mechanistic insight” or “interesting, but an insufficient advance” and saved them countless hours of additional experiments thought up by reviewers simply to increase the ever-elusive “impact” of the work. Since its launch, BiO has published more than 450 freely accessible articles.

## Turn-around time and quality of review

One of the foremost objectives of BiO was to address the speed and quality of review. Review procedures at BiO are fast and simple, but also rigorous. We ask reviewers to complete their reviews within seven working days, and our average time for completing peer review in 2014 was nine days. Almost no articles are accepted without some changes being requested, but new experiments are only insisted upon if these are essential to support the key conclusions of the paper; we do not ask our reviewers to suggest experiments that would simply increase the impact of the work. Articles are published online on average 25 working days after acceptance.

## Reviewer fatigue

The multiple rounds of review, revision and resubmission often required before a paper is finally accepted has led to substantial reviewer fatigue, with large numbers of reviewers often involved with the peer review process for a single article. BiO accepts articles and any accompanying reviews from the other Company of Biologists' journals. In addition, we have an agreement in place with *eLife*, the journal published by the Wellcome Trust, Howard Hughes Medical Institute and the Max Planck Society, whereby their rejected authors are given the opportunity to transfer to BiO. We are negotiating similar deals with other journals. We will also consider peer review reports from other journals. Such transfer of reviews and articles speeds up editorial decisions and reduces the number of rounds of review.

## Ethical publication

Publishers have an increasing array of tools to help identify possible unethical practices, such as the iThenticate software, which detects plagiarism, and processes that reveal potential figure manipulation. Recently, the increasing number of reports of ‘fake’ reviews, whereby authors manipulate the review process to enable them to review their own work (http://www.nature.com/news/publishing-the-peer-review-scam-1.16400), has led us to review our own procedures. We will ensure that any author-suggested reviewers with a non-institutional e-mail address (such as Gmail or Yahoo) are indeed valid and associated with a ‘real’ scientist. Would publishing peer reviews eradicate weak or non-existent peer review practices adopted by so-called predatory publishers (http://scholarlyoa.com/publishers/), who seem to publish anything for a fee? We continue to be actively involved in this debate with other reputable publishers.

## Recognition and reward

Many publishers are wrangling with the issue of reviewer support, reward and incentive. Increasing numbers of articles are being published and the demand for peer review has never been greater. How is the quality of peer review to be maintained in such an environment? Are senior investigators able to dedicate their valuable time to mentoring early career scientists in the art of peer review? BiO encourages the involvement of postdocs and other early career scientists in the peer review process. We simply ask that: the name of the co-reviewer is reported to the Editor; the same rules of confidentiality and conflict of interest be applied; there is a genuine mentoring process; and the senior invited reviewer takes responsibility for the report delivered to the journal. Taking on the role of reviewer is usually not acknowledged by institutes and funders as a research output, but it is a cornerstone of the publication process. We continue to campaign with other publishers for this vital service to be properly recognized as an important academic contribution. Should reviewers be paid or is there in effect a payment in kind, an altruistic understanding that acting as a peer reviewer supports the community? This conversation will continue, with journals undertaking experiments to improve or refine the peer review process, and investigating ways to reward peer reviewers. Today, as we do every year, we take a small step in this direction by publishing the names of all those who completed a review for BiO during 2014, with our sincere thanks for their expertise and time. We hope the community will join us in thanking these people properly for their considerable effort.

Alejandro Aballay, Duke University, USA

Peter Aerts, University of Antwerp, Belgium

Kami Ahmad, Harvard Medical School, USA

Takahiko Akematsu, York University, Toronto, Canada

Scott Alper, National Jewish Health, USA

Jeffrey Amack, SUNY Upstate Medical University, USA

Enrique Amaya, University of Manchester, UK

Jack Arbiser, Emory University, USA

Maria Eugenia Arnone, Stazione Zoologica Anton Dohrn, Italy

Damien Arnoult, INSERM U542, France

Kristin Artinger, University of Colorado, USA

Jerome Artus, Pasteur Institut, France

Sophie Astrof, Weill Medical College of Cornell University, USA

Liliana Attisano, University of Toronto, Canada

Peter Baas, Drexel University College of Medicine, USA

Luis-Alberto Baena-Lopez, MRC National Institute for Medical Research, UK

Mohan Balasubramanian, University of Warwick, UK

Brian Ballios, University of Toronto, Canada

Allan Balmain, University of California San Francisco, USA

George Banting, University of Bristol, UK

Jeffrey Barminko, Rutgers University, USA

Mark Bass, University of Bristol, UK

Andrew Bassett, MRC Functional Genomics Unit, University of Oxford, UK

Aaron Baumann, Janelia Research Campus, Howard Hughes Medical Institute, USA

Renaud Beauwens, Université libre de Bruxelles, Belgium

Catherina Becker, University of Edinburgh, UK

Frederic Becq, University of Poitiers, France

Christine Bedore, Duke University, USA

Tracy Bedrosian, Ohio State University, USA

Frank Beier, University of Western Ontario, Canada

Joseph Bell, Harvard Medical School, USA

Igal Berenshtein, Ben-Gurion University of the Negev, Israel

Andreas Bergmann, University of Massachusetts Medical School, USA

Anne Bertolotti, MRC Laboratory of Molecular Biology, Cambridge, UK

David Bilder, University of California Berkeley, USA

Joyce Bischoff, Boston Children's Hospital, USA

Jorge Blanco, Institute of Population Genetics, Vienna, Austria

Bruce Blumberg, University of California Irvine, USA

Rachel Bowden, Illinois State University, USA

Douglas Bowles, University of Missouri, USA

Andre Brandli, Ludwig-Maximilian University of Munich, Germany

Beate Brand-Saberi, Ruhr Universitat Bochum, Germany

Björn Brembs, Universität Regensburg, Germany

Josh Brickman, University of Edinburgh, UK

Steven Britt, University of Colorado at Denver and Health Sciences Center, USA

William Brownell, Baylor College of Medicine, USA

Nia Bryant, University of York, UK

Bradley Buckley, Portland State University, USA

Marina Campione, CNR Institute of Neurosciences, Italy

Dana Carroll, University of Utah, USA

Tamara Caspary, Emory University, USA

Kevin Chalut, University of Cambridge, UK

Luke Chamberlain, Strathclyde University, UK

Yu Chen, National Institutes of Health, Bethesda, USA

Linxi Chen, University of South China, China

Xie Jie Chen, SUNY Upstate Medical University, USA

Danian Chen, Lunenfeld-Tanenbaum Research Institute, Mount Sinai Hospital, Toronto, Canada

Lionel Christiaen, New York University, USA

Christopher Ciarleglio, Brown University, USA

Lionel Clement, University of Alabama School of Medicine, USA

David Clynes, Weatherall Institute of Molecular Medicine, Oxford, UK

Thomas Coate, Georgetown University, USA

Raymond Cohen, CSIRO, Australia

Paola Costantini, University of Padova, Italy

Ita Costello, University of Oxford, UK

Matthew Cottee, University of Oxford, UK

Evelyne Coudrier, Institut Curie, France

Fraser Coxon, Bone Research Society, UK

Julie Crockett, University of Aberdeen, UK

Robyn Crook, University of Texas Health Science Center at Houston, USA

Vincent Croset, University of Oxford, UK

Colin Crump, University of Cambridge, UK

Andrew Dacks, West Virginia University, USA

Richard Dahl, Indiana University, USA

Kaveh Daneshvar, Massachusetts General Hospital, Harvard Medical School, USA

Peter Daniel, Hofstra University, USA

Kristiaan D'Aout, University of Liverpool, UK

Stefano De Renzis, EMBL Heidelberg, Germany

Johan De Rooij, Hubrecht Institute, The Netherlands

Pascal de Santa Barbara, INSERM U1046, Montpellier, France

Jean-Luc Desseyn, Université de Lille, France

Julie Donaldson, NIH, Bethesda, USA

Mary Donohoe, Cornell University, USA

Cynthia Downs, University of Nevada Reno, USA

Dennis Drescher, Wayne State University, USA

Jeoffrey Eales, University of Manitoba, Canada

John Eggenschwiler, University of Georgia, USA

Peter Falkingham, Royal Veterinary College, UK

Alan Fanning, The University of North Carolina at Chapel Hill, USA

David Fay, University of Wyoming, USA

Brock Fenton, University of Western Ontario, Canada

Rafael Fissore, University of Massachusetts, USA

Ann Foley, Clemson University College of Engineering, USA

Eric Folker, Boston College, USA

Caroline Formstone, Kings College London, UK

Amelie Fradet-Turcotte, The Lunenfeld-Tanenbaum Research Institute, Mount Sinai Hospital, Toronto, Canada

Diego Franco, University of Jaén, Spain

Alison Frand, David Geffen School of Medicine at UCLA, USA

Vanessa Franssens, KU Leuven, Belgium

Mariella Freitas, University of Viçosa, Brazil

Thomas Friedman, NIDCD, National Institutes of Health, Bethesda, USA

Mark Frye, University of California, Los Angeles, USA

Christopher Fulton, Australian National University, Australia

Antonella Galli, Wellcome Trust Sanger Institute, UK

Brigitte Galliot, University of Geneva, Switzerland

Deborah Galson, University of Pittsburgh, USA

Michael Garcia, University of Missouri-Columbia, USA

Alexis Gautreau, École Polytechnique, France

Elizabeth Gavis, Princeton University, USA

Charles Gerday, University of Liege, Belgium

Troy Ghashghaei, North Carolina State University, USA

Marta Giacomello, Dulbecco-Telethon Institute, Italy

Allen Gibbs, University of Nevada, USA

Gary Gillis, Mount Holyoke College, USA

Fernando Giraldez, Universitat Pompeu Fabra, Spain

Mary Goll, Memorial Sloan Kettering Cancer Center, USA

Sonia González-Braojos, Museo Nacional de Ciencias Naturales, Madrid, Spain

Andrew Grierson, University of Sheffield, UK

Atan Gross, Weizmann Institute of Science, Israel

Francois Guillemot, National Institute for Medical Research, UK

Monika Gullerova, University of Oxford, UK

Ufuk Günesdogan, University of Cambridge, UK

Marina Guvakova, University of Pennsylvania, USA

Randal Halfmann, University of Texas Southwestern Medical Center, USA

Chrissy Hammond, University of Bristol, UK

Steven Hann, Vanderbilt-Ingram Cancer Center, USA

Jeffrey Harrison, University of Florida, USA

Volker Hartenstein, UCLA, USA

Simon Harvey, Canterbury Christ Church University, UK

Adrian Harwood, UCL, UK

Philip Hastings, Baylor College of Medicine, USA

Harry Heijnen, University Medical Center Utrecht, The Netherlands

Ian Henderson, University of Cambridge, UK

Brian Hendrich, University of Cambridge, UK

Clarissa Henry, University of Maine, USA

Ian Hickson, University of Copenhagen, Denmark

Helfrid Hochegger, University of Sussex, UK

Peter Hohenstein, MRC Human Genetics Unit, UK

Georg Hollander, University of Oxford, UK

Edmund Hollis, UCSD, USA

Pamela Hoodless, BC Cancer Agency, Canada

Sevan Hopyan, The Hospital for Sick Children, Toronto, Canada

Dan Hultmark, Umeå University, Sweden

Peter Hurd, University of Alberta, Canada

Richard Hynes, HHMI/MIT, USA

Hiroshi Imai, Kyoto University, Japan

Luisa Iruela-Arispe, UCLA, USA

John Isanhart, US Department of the Interior, USA

Shoko Ishibashi, University of Manchester, UK

Yoshifumi Itoh, University of Oxford, UK

Aymelt Itzen, Technische Universität München, Germany

Juan Carlos Izpisua Belmonte, Salk Institute, USA

Catherine Jackson, Institut Jacques Monod, CNRS, France

Abhinav Jain, MD Anderson Cancer Center, USA

Jorgen Jensen, Norwegian School of Sport Sciences, Oslo, Norway

Renjie Jiao, Institute of Biophysics, Chinese Academy of Sciences, China

Mats Johansson, University of Wisconsin, USA

Reid Johnson, University of California, USA

Marko Kaksonen, EMBL Heidelberg, Germany

David Kang, USF Health Byrd Alzheimer's Institute, USA

Laurent Keller, University of Lausanne, Switzerland

Steven Kelly, University of Oxford, UK

Anna-Marie Kenney, Emory University, USA

Yoshiaki Kikkawa, Tokyo Metropolitan Institute of Medical Science, Japan

Dennis Kim, MIT, USA

Chungho Kim, Korea University, Republic of Korea

James Konopka, Stony Brook University, USA

Edward Korn, NHLBI, NIH, USA

Vladimir Korzh, Institute of Molecular and Cell Biology, Singapore

Helmut Kramer, University of Texas Southwestern Medical Center, USA

Scott Kreher, Dominican University, USA

Torsten Kristensen, Aalborg University, Denmark

Ronald Kroger, Lund University, Sweden

Paul Kulesa, Stowers Institute for Medical Research, USA

Diana Laird, UCSF, USA

Pavithra Lakshminarasimhan, University of Cambridge, UK

Fredrik Lanner, Karolinska Institutet, Sweden

Michael Lanzer, Heidelberg University Hospital, Germany

Miguel Leal, Universidade de Aveiro, Portugal

Jae Lee, University of Miami, USA

Heon-Jin Lee, Kyungpook National University, Republic of Korea

Emilie Legue, Yale University, USA

Jinxing Lin, Institute of Botany, Chinese Academy of Sciences, China

Ji-Long Liu, MRC Functional Genomics Unit, University of Oxford, UK

Na Liu, Nankai University, China

Ying Liu, University of Texas Health Science Center at Houston, USA

Aimin Liu, Penn State University, USA

Catherine Lohmann, University of North Carolina, USA

Martin Lowe, University of Manchester, UK

Sally Lowell, University of Edinburgh, UK

Laura Machesky, CRUK Beatson Institute for Cancer Research, Glasgow, UK

Yusuke Maeda, Osaka University, Japan

Eamonn Mallon, University of Leicester, UK

Marcos Malumbres, Centro Nacional de Investigaciones Oncologicas, Madrid, Spain

Ivan Manzini, University of Göttingen, Germany

Giancarlo Marra, University of Zurich, Switzerland

Jennifer Mather, University of Lethbridge, Alberta, Canada

Ivan Matic, U1001 INSERM, France

Michiyuki Matsuda, Kyoto University, Japan

Takeshi Matsuzawa, Osaka Prefecture University, Japan

Heidi McBride, McGill University, Canada

David McClay, Duke University, USA

Mark McNiven, Mayo Clinic, USA

Irene Miguel-Aliaga, Imperial College London, UK

Thomas Millard, University of Manchester, UK

Laura Miller, University of North Carolina - Chapel Hill, USA

Lucile Miquerol, Aix-Marseille University, CNRS UMR 7288, France

Toshihiro Mitaka, Sapporo Medical University School of Medicine, Japan

Christian Mitri, Institut Pasteur, France

Miguel Moreno-Garcia, Colorado State University, USA

Ciaran Morrison, National University of Ireland, Galway, Ireland

Scott Moye-Rowley, University of Iowa, USA

Hans-Arno Muller, University of Dundee, UK

Daniel Mulvihill, University of Kent, UK

Jan Munch, Ulm University Hospital, Germany

Silvia Munoz-Descalzo, University of Bath, UK

Sean Munro, MRC Laboratory of Molecular Biology, Cambridge, UK

Nagy Nandor, Semmelweis University, Budapest, Hungary

Anthony Nicholas, University of Alabama School of Medicine, USA

Jennifer Nichols, University of Cambridge, UK

Ben Nichols, MRC Laboratory of Molecular Biology, Cambridge, UK

Roland Nitschke, Life Imaging Center, Freiburg, Germany

Ryusuke Niwa, University of Tsukuba, Japan

Wataru Nomura, Tokyo Medical and Dental University, Japan

Akinao Nose, University of Tokyo, Japan

Scott Nowak, Kennesaw State University, USA

Sonja Nowotschin, Memorial Sloan-Kettering Cancer Center, USA

Niels Ortenblad, University of Southern Denmark, Denmark

James Palis, University of Rochester Medical Center, USA

Yale Passamaneck, University of Hawaii, USA

Andrew Peden, University of Sheffield, UK

Giuseppa Pennetta, University of Edinburgh, UK

Jonathan Pettitt, University of Aberdeen, UK

Cathie Pfleger, Mount Sinai School of Medicine, USA

Anna Philpott, University of Cambridge, UK

Jonathon Pines, University of Cambridge, UK

Nicolas Pollet, ISSB, CR1 CNRS, France

Yekaterina Poloz, University of Toronto, Canada

Herman Pontzer, Hunter College, USA

Jean Potvin, Saint Louis University, USA

Rytis Prekeris, University of Colorado Health Sciences Center, USA

John Presley, University of Montreal, Canada

Victoria Prince, University of Chicago, USA

Ruben Quintana-Cabrera, University of Padova, Italy

R Raijmakers, University of Utrecht, The Netherlands

Amy Ralston, Michigan State University, USA

V Krishnan Ramanujan, Cedars-Sinai Medical Center, USA

Mary Ramsey, University of Texas, USA

Roberto Refinetti, University of South Carolina, USA

Evan Reid, Cambridge Institute for Medical Research, UK

Heinrich Reinchert, University of Basel, Switzerland

Victor Rizzo, Temple University School of Medicine, USA

Hugh Robertson, University of Illinois at Urbana-Champaign, USA

Tristan Rodriguez, MRC Clinical Sciences Centre, Imperial College London, UK

Shai Sabbah, Brown University, USA

Alvaro Sagasti, UCLA, USA

Aki Salo, University of Bath, UK

Emilio Salo, Universitat de Barcelona, Spain

Ramkumar Sambasivan, inStem, India

Lisa Sandell, University of Louisville, USA

Pauline Schaap, University of Dundee, UK

Michael Schaller, West Virginia University, USA

Andreas Schedl, Institute of Biology Valrose, France

Richard Sherwood, Brigham and Women's Hospital, USA

Yun-Bo Shi, NICHD/NIH, Bethesda, USA

Thierry Soldati, University of Geneva, Switzerland

Maria Eugenia Soriano, Dulbecco Telethon Institute, Italy

Shankar Srinivas, University of Oxford, UK

Michelle Starz-Gaiano, University of Maryland, USA

Michelle Staudinger, University of Massachusetts, USA

Jared Sterneckert, Max Planck Institute for Molecular Biomedicine, Germany

Angelika Stollewerk, Queen Mary University of London, UK

Wenjing Su, Memorial Sloan-Kettering Cancer Center, USA

Christine Suetterlin, University of California-Irvine, USA

Melanie Sutton-McDowall, University of Adelaide, Australia

Tohru Suzuki, Tohoku University, Japan

Markus Tamas, University of Gothenburg, Sweden

Kayoko Tanaka, University of Leicester, UK

Andrea Tedeschi, German Center for Neurodegenerative Diseases, Germany

William Theurkauf, University of Massachusetts Medical School, USA

Ulrich Thomas, Leibniz Institute for Neurobiology, Germany

Ryan Thummel, Wayne State University, USA

Andrzej Tkacz, University of Oxford, UK

Sokol Todi, Wayne State University, USA

Stan Tomavo, Université Lille Nord de France, France

Mark Tomishima, Memorial Sloan Kettering Cancer Center, USA

Michael Tomlinson, University of Birmingham, UK

Pranav Ullal, University of Lausanne, Switzerland

Teresa Valencak, Veterinary University Vienna, Austria

Mark van Breugel, MRC Laboratory of Molecular Biology, Cambridge, UK

Anne Marijn van der Graaf, University of Groningen, The Netherlands

Chris van der Poel, La Trobe University, Australia

Frans van Roy, Ghent University, Belgium

Alexander Venn, Centre Scientifique de Monaco, Monaco

Monica Vetter, University of Utah, USA

Andrea Vortkamp, Max Planck Institute for Developmental Biology, Germany

Stephen Voss, University of Kentucky, USA

James Wakefield, University of Exeter, UK

Yibin Wang, UCLA, USA

Xiaoxi Wang, Vanderbilt University, USA

Hongyan Wang, Duke-NUS Graduate Medical School, Singapore

Steven Wasserman, UCSD, USA

Thomas Wassmer, Aston University, UK

Thomas Wellems, NIAID, NIH, USA

Michael White, University of South Florida, USA

Jill Wildonger, University of Wisconsin-Madison, USA

Bodo Wilts, University of Fribourg, Switzerland

Richard Wingate, King's College London, UK

Thomas Woolley, University of Oxford, UK

Christopher Wright, Vanderbilt University, USA

Wei-Feng Xue, University of Kent, UK

Zhe Yang, Wayne State University, USA

Seda Yerlikaya, University of Geneva, Switzerland

Hebao Yuan, University of Michigan, USA

Troy Zars, University of Missouri-Columbia, USA

Bing Zhang, University of Missouri, USA

Hong-Bo Zhao, University of Kentucky Medical Center, USA

Hui Zhao, The Chinese University of Hong Kong, Hong Kong

Irene Zohn, Children's National Medical Center, USA

Olivier Zugasti, Centre d'Immunologie de Marseille-Luminy, France

